# Prevalence of the Usage of Smokeless Tobacco in Patients Presenting With Stroke in a Tertiary Care Hospital

**DOI:** 10.7759/cureus.21060

**Published:** 2022-01-09

**Authors:** Zainab Saleem, Sumayyah Liaquat, Mohsina Syed, Zaira Abbas, Amani Amir, Naila N Shahbaz

**Affiliations:** 1 Neurology, Dow University of Health Sciences, Civil Hospital Karachi, Karachi, PAK; 2 Stroke Medicine, Blackpool Teaching Hospitals NHS Foundation Trust, Blackpool, GBR; 3 Internal Medicine, Hussain Lakhani Hospital, Karachi, PAK; 4 Neurology, Dow University of Health Sciences, Karachi, PAK

**Keywords:** karachi, gutka, smokeless tobacco, paan, stroke

## Abstract

Background

Stroke is one of the leading causes of disability, long-term morbidity, and mortality. The key modifiable risk factors for stroke are largely driven by demographic changes and various social adaptations. Smokeless tobacco consumption is high in developing countries with less awareness of its potential health risks.

Aim

This study was designed to determine the prevalence of usage of smokeless tobacco in patients presenting with stroke in a tertiary care hospital in Karachi.

Methods

This is a cross-sectional study conducted at the Department of Neurology of Dr. Ruth K. M. Pfau Civil Hospital in Karachi. A total of 192 patients were enrolled during the study period of six months, from September 2018 to March 2019. All consenting patients were recruited who presented with stroke and fulfilled the inclusion criteria. All patients were assessed by a trained neurologist.

Results

A total of 192 patients with stroke were included. There were 146 (76%) males and 46 (24%) females with a mean age of 53 ± 6.1 years. The highest percentage (39%) of cases was illiterate. A majority (64%) of patients presented belonged to a lower socioeconomic background. Out of 192 stroke patients, 131 (68.2%) consumed smokeless tobacco.

Conclusion

The frequency of smokeless tobacco consumption was found to be high in stroke patients who require the attention of the physician on modifiable risk factors.

## Introduction

In recent years, the usage of smokeless tobacco has soared in developing countries [1]. Smokeless tobacco use is most popular in certain geographical locations, including India, Pakistan, Bangladesh, South Asia, and in certain parts of Africa [2]. In Pakistan, the prevalence in adolescence ranged from 16.1 percent to 20 percent [3].

A strong link exists between cigarettes and stroke [4]. However, the association between smokeless tobacco and stroke risk has not been well-established in the literature. Smoking increases the cerebrovascular risk two to four times among men and women [4]. In a study conducted in Sweden, where snuff consumption is widespread, current snuff users had a higher risk of fatal ischemic stroke than non-users [5]. Ruchika Gupta et al. carried out a systematic review to examine the relationship between smokeless tobacco and cardiovascular disease and found inadequate evidence to establish the link [6]. Whereas, a systematic review with meta-analysis was done in 2009 by Paola et al. concluded that there is an increased risk of fatal myocardial infarction and stroke among users of smokeless tobacco compared to non-users [7].

The primary goal of this study was to determine the prevalence of smokeless tobacco usage in stroke patients in our populace, where smokeless tobacco consumption is significantly more widespread, predominantly in the form of paan and gutka. More research should be done in the future to see if there is a causal association between smokeless tobacco and stroke.

## Materials and methods

A cross-sectional study of six months duration was performed in the Department of Neurology of Dr. Ruth K. M. Pfau Civil Hospital in Karachi, Pakistan, from September 2018 to March 2019. The study was authorized by the Civil Hospital Karachi's ethics committee and the Head of the Department of Neurology. All patients who were involved in the study gave informed and written consent. The study included both males and females between the ages of 30 and 60 years, and both ischemic and hemorrhagic stroke types were included. The CT scan was done initially to rule out bleeding, and in patients whose CT came back normal, an MRI brain stroke protocol was done to look for ischemia. Patients who had a space-occupying lesion or intracranial tumor were excluded from the study. Also, patients who had a history of alcohol addiction and drug use, patients who had cerebral venous sinus thrombosis, meningitis, encephalitis, demyelinating lesions, or vasculitis were excluded. 

Smokeless tobacco users were defined as using paan or gutka or both at present. A cigarette smoker was defined as having smoked more than one pack per year. Hypertension was defined as raised blood pressure, blood pressure (BP) >140/90 mmHg, past history of hypertension, or history of use of antihypertensive drugs. Diabetes mellitus was defined by the American Diabetes Association diagnostic criteria (a fasting plasma glucose (FPG) level of 126 mg/dL or higher, or a random plasma glucose of 200 mg/dL or higher in a patient with classic symptoms of hyperglycemia) [8]. Dyslipidemia was defined according to the American Heart Associations' classification as total cholesterol 200 mg/dl, low-density lipoprotein (LDL) 130 mg/dl, high-density lipoprotein (HDL) 35 mg/dl, or a combination thereof [9]. Socioeconomic status was defined as lower (income per capita 20000), middle (income per capita 20000-50000), and upper class (income per capita > 50000). 

​​​​​On a predesigned proforma, detailed history and demographic information, use of smokeless tobacco, other comorbidities were entered. The history of patients with aphasia was obtained by their attendants. All the data was collected by neurologists in training.

Data was analyzed on SPSS version 20 (IBM Inc., Armonk, New York). The mean and standard deviation were calculated for a quantitative variable like age, duration of a stroke, and duration of smokeless tobacco use. Frequency and percentages were calculated for qualitative variables such as employment status, educational level, socioeconomic status, type of stroke, diabetes mellitus, dyslipidemia, hypertension, duration of stroke, smokeless tobacco use, and type of smokeless tobacco.

## Results

The study comprised a total of 192 patients. There were 146 (76%) male and 46 (24%) female patients. The mean age of the sample was 53 (±6.1) years.

A total of 94 (49%) subjects were employed, and 98 (51%) were unemployed at the time of onset of stroke. The highest percentage (39.6%) of cases were illiterate, while only 14.1% had higher education. A majority (64.6%) of patients presented belonged to a lower socioeconomic background (Table 1).

**Table 1:** Sample characteristics

Variables	%(n) [n=192]
Age (years), mean ± SD	53±6.1
Gender	
Male	76 (146)
Female	24 (46)
Educational level	
Illiterate	39.6 (76)
Primary	24.5 (47)
Secondary	21.9 (42)
Higher	14.1 (27)
Employment status	
Employed	49 (94)
Unemployed	51 (98)
Socioeconomic status	
Lower	64.6 (124)
Middle	27.1 (62)
Higher	8.3 (15)

In the overall study population, 137 (71.4%) of patients had an ischemic stroke, and 55 (28.6%) had a hemorrhagic stroke. A total of 109 (56.8%) subjects presented within 24 hours of stroke onset, whereas 83 (43.2%) arrived at the hospital late. At the time of the presentation, 131 (68.2%) were using smokeless tobacco. Moreover, 63 (32.8%) used paan on a regular basis, whereas 57 (29.7%) consumed gutka. Both sorts of smokeless tobacco were used by 11 (5.7%).

The majority of patients, 109 (56.8%), had been taking smokeless tobacco for more than 10 years prior to their stroke presentation. Concurrent smoking was practiced by 37 (19.3%) patients (Table 2).

**Table 2:** Association of stroke with smokeless tobacco usage

Variables	%n [n=192]
Type of stroke	
Ischemic	71.4 (137)
Hemorrhagic	28.6 (55)
Time of presentation	
Within 24 hours	56.8 (109)
After 24 hours	43.2 (83)
Use of smokeless tobacco	68.2 (131)
Type of smokeless tobacco	
Paan	32.8 (63)
Gutka	29.7 (57)
Both	5.7 (11)
Duration of smokeless tobacco use	
>10 years	56.8 (109)
<10 years	43.2 (83)

Other stroke risk factors identified in these patients were hypertension, dyslipidemia and diabetes mellitus in 77.6%, 66.7% and 34.9%, respectively.

## Discussion

According to this study, around 68% of stroke patients had a history of smokeless tobacco usage. The incidence of smokeless tobacco usage was higher among persons from lower socioeconomic backgrounds (64.6%) and those who were illiterate (39.6%) and unemployed (51%). It is assumed that the lower socioeconomic status of the population has a higher prevalence because of its minimal cost. People who are illiterate are unaware of the potentially catastrophic effects of using it. Previous research from Pakistan [10,11] and India [12,13] has found an inverse relationship between smokeless tobacco usage and socioeconomic status and education level.

In Pakistan (16-20%) and India (20%), the prevalence of smokeless tobacco is nearly identical [3,14]. Jena et al. had the highest number of males (77.7%) [14]. In our study, consumption of smokeless tobacco was also higher in males than in females (76% vs. 24%).

Various types of smokeless tobacco

Smokeless tobacco comes in a variety of forms, including; paan, which is a mixture of areca nut, betel nut, tobacco, and slaked lime, which is wrapped in a betel leaf. Areca nut coated with a tobacco powder is known as gutka. Naswar is a moist, powdered tobacco dip with added flavoring agents. Pulverized dried leaves of tobacco mixed with slaked lime is khaini, a tobacco and sugar molasses paste is known as gudakhu, iqmik (punk ash) is a mixture of tobacco and the ash of* phellinus igniarus*, and mishri is burned tobacco [15]. It is consumed by either rubbing it inside the mouth or chewed, and some forms are snuffed or sucked by users.

The type of smokeless tobacco differs depending on the locale. In South Asia, for example, betel nut is most widely used. The most common types of smokeless tobacco used in Pakistan are paan, gukta, and naswaar. In Karachi, paan and gukta are the predominant type of smokeless tobacco which is easily available and is low cost, whereas naswaar is a common type in Khyber Pakhtunkhwa (KPK). Paan, gutka, and betel nut were more popular among Hazara and north Punjab migrants, whereas naswaar was the favored smokeless tobacco among Pathans from the Khyber Pakhtunkhwa province, according to an Iqbal et al.'s study [4]. Paan users were found to be significantly higher (32.8%) than gutka users (29.7%) in this study. Paan (38.9%) was also the most preferred form of smokeless tobacco in Jena et al.'s study [14]. The majority of subjects in our study were using smokeless tobacco for more than 10 years (56.8%).

The most prevalent type of stroke was observed to be ischemic stroke [71.4%] in this study. Ischemic stroke was also the most common type of stroke in a study by Jena et al. [14]. One Indian study showed that chewing tobacco increases the risk of cardiovascular disease [16].

Smokeless tobacco produces the largest levels of nicotine in the blood, which causes an increase in sympathetic drive, which can lead to elevated blood pressure [17]. In our study, hypertension, diabetes mellitus, and dyslipidemias were found in 77.6%, 34.9%, and 66.7%, respectively. A study by Saba Zaidi demonstrated hypertension in 81.5%, diabetes mellitus in 75.6%, dyslipidemias in 63% of patients of acute ischemic stroke, but it only included young patients (20-49 years ) with acute ischemic stroke and smokeless tobacco usage (daily for past two years) was found to be 28.15 % [18]. 

Out of 128 (66.7%) patients with dyslipidemia, 88 (45.8%) patients had elevated LDL-cholesterol (C), and HDL-C was impaired in 50 (26%) patients. No significant association was observed between smokeless tobacco usage and dyslipidemia (p-value=0.66).

## Conclusions

Tobacco use is a preventable cause of stroke. Warnings and education about smokeless tobacco should be presented in the same way that they are for smoking. More widespread research on greater sample size is needed to delineate the mechanisms of smokeless tobacco on the cardiovascular system and its association with stroke.
